# OR2-001 – The possible role of pyrin on cell migration

**DOI:** 10.1186/1546-0096-11-S1-A1

**Published:** 2013-11-08

**Authors:** B Peynircioğlu, ZY Akkaya, C Guler, A Cetinkaya, ZE Taskiran, E Yilmaz

**Affiliations:** 1 Medical Biology, Hacettepe University, Ankara, Turkey; 2 Medical Genetics, Hacettepe University, Ankara, Turkey

## Introduction

MEFV which encodes pyrin, cause familial Mediterranean fever (FMF), the most common auto-inflammatory disease. The pyrin protein appears to be a regulator of inflammation, but its exact role on inflammatory pathways is still controversial. Several pyrin-interacting proteins have been identified, each of which are related to inflammation through regulation of cell death, cytokine secretion, and cytoskeletal signaling. It has been documented that in migrating human monocytes, pyrin protein is dramatically polarized at the leading edge, where it co-localizes with polymerizing actin. Thus, we hypothesized that pyrin may have a key role in cell migration through its interaction with well-known regulators of inflammatory cell migration.

## Objectives

In this study we aimed to examine more closely the distribution of pyrin and possible pyrin-related proteins that has cytoskeletal functions through actin machinery such as; LSP1(Leukocyte-specific protein 1), PSTPIP2 (Proline-serine-threonine phosphatase interacting protein 2), LPXN(Leupaxin), DAAM1(Dishevelled associated activator of morphogenesis 1, WDR1(WD repeat containing protein 1), and WIPF3 (WAS/WASL-interacting protein family member 3) in migrating cells.

## Methods

Firstly; wound healing assay was used to show the effect of pyrin in transiently transfected HeLa cells and Boyden chamber assays were usedin order to quantitate this effect in transiently transfected COS-7 cells migrating against an insulin gradient. Then the distribution and expression of pyrin and actin related proteins were analyzed during cell migration process using a functional HL-60 cell migration assay. qRT-PCR was performed in order to analyze the expression profile of actin related proteins during cell migration. Then IF (immunofluorescence) staining technique was used to demonstrate the cellular distribution of these proteins.

## Results

A significant relationship between wild type pyrin overexpression and increased cell migration was shown both in Boyden chamber and wound healing assays. Preliminary results related with HL-60 cell migration assay showed that expression levels of LSP1, LPXN and WDR1 change during migration but not significantly; while PSTPIP2, DAAM and WIPF3 have a very low basal expression level in these cells. Interestingly; IF co-stainings showed that pyrin and these proteins co-localize at the leading edge of the migrating cells, where actin polymerization occurs.

## Conclusion

These studies described here provide a new insight to the potential role of pyrin protein in the process of cell migration. We have demonstrated that the regulation of pyrin and its interacting partners during cell migration occurs post-translationally rather than transcriptional level. Further studies on; i) RNA interference mechanism targeting MEFV expression and ii) LPS treatment during cell migration are underway and may lead to understand the exact role of pyrin in inflammatory cells and result in novel treatment options for inflammatory diseases in general.

This study was supported by The Technical and Scientific Research Council of Turkey (TUBITAK) Project Number: TUBITAK 1001-SBAG-111S507

## Disclosure of interest

None declared.

